# scIRT: Imputation and Dimensionality Reduction for Single-Cell RNA-Seq Data by Combining NMF with SMOTE

**DOI:** 10.3390/ijms27031173

**Published:** 2026-01-23

**Authors:** Yunwen Mou, Shuchao Li, Guoli Ji

**Affiliations:** Department of Automation, Xiamen University, Xiamen 361005, China; monyunweng@stu.xmu.edu.cn (Y.M.); sclee@stu.xmu.edu.cn (S.L.)

**Keywords:** single-cell RNA-seq data, dropout, NMF, SMOTE, clustering

## Abstract

The establishment and development of single-cell RNA-sequencing (scRNA-seq) technology has accelerated the analysis of cell genome characteristics down to the single-cell level. Despite the rapid development of scRNA-seq technology, we cannot obtain a complete gene expression matrix in the biological experiments, and the scRNA-seq data obtained from experiments also have a high dropout rate. Unfortunately, gene expression analysis and clustering tools require a complete matrix of gene expression values for classification or clustering calculations. Most imputation methods focus on the impact of the imputed high-dimensional expression matrix on clustering and cannot obtain the low-dimensional representation matrix, which may have an even better guiding effect on clustering. To this end, we designed an iterative imputation pipeline called scIRT to estimate dropout events for scRNA-seq and achieve dimensionality reduction simultaneously by combining the synthetic minority over-sampling technique (SMOTE) and non-negative matrix factorization (NMF). The adaptation of SMOTE effectively imputes missing data, while NMF performs dimensionality reduction and feature extraction on high-dimensional data. Using several scRNA-seq datasets, we demonstrated that this new approach achieved better and more robust performance than the existing approaches. We also compared the different effects of the imputed matrix and the low-dimensional representation matrix on clustering. ScIRT is a tool that can be used to preprocess scRNA-seq data. It can effectively recover missing data from scRNA-seq to facilitate downstream analyses such as cell type clustering and visualization.

## 1. Introduction

Single-cell RNA sequencing (scRNA-seq) is rapidly becoming an efficient and robust tool for analyzing the genome-scale transcription from a single cell and capturing cell-to-cell variability within the cell transcriptome [1]. With the continuous advancement of the latest technology, a large number of different scRNA-seq datasets are being generated, which can be used to hierarchically cluster cells [2], determine cell types and states [3], reconstruct the developmental trajectory of cells [4], and identify the key genes involved in the cell life cycle [5]. A large amount of scRNA-seq data makes it possible to analyze the diverse and heterogeneous structure of cells. However, due to the low efficiency of mRNA capture, scRNA-seq technology suffers from biological noise during the transcription process [6]. Although great progress has been made in the field of single-cell sequencing analysis, we still face many challenges in this field, one of which is the so-called ‘dropout’ event. The ‘dropout’ phenomenon refers to the extremely high variability of the single-cell expression profile of the gene in the process of transcription and the loss of a large proportion of the data in the expression matrix (typically 80% and the limit reaches 95%), which hinders the reliability of gene expression, especially for the low or moderate expression in different cells, resulting in an extremely sparse gene-cell expression matrix and hindering downstream biological analysis [7]. Therefore, there is an urgent need to develop new approaches to modify the expression level of genes in scRNA-seq data.

To estimate the missing values in the scRNA-seq data, a large number of approaches have been proposed. MAGIC [8] is the first approach to impute missing data from scRNA-seq at the single-cell level. It is a Markov affinity method based on the thermal diffusion view that estimates unobserved expression events by pooling data for each gene in similar cells. ScImpute [9] estimates the dropout event of each gene in the sample through a mixed model. More advanced than MAGIC, scImpute can identify the authenticity of missing values by distinguishing which zeros are missing values and which zeros are true values. Unlike MAGIC and scImpute, SAVER [10] uses the Bayesian method to recover the true expression level of the gene in the same cell, using the binomial distribution to replace the original gene expression. The advantage of SAVER over MAGIC and scImpute is that it provides a measure of uncertainty for the recovery of missing values in scRNA-seq data. Both MAGIC and SAVER changed the global gene expression level, including genes that are not affected by the dropout event, so global adjustment can introduce new errors into the data. There are several approaches to reducing errors by clustering similar cells. For example, drImpute [11] uses different cell clustering results to perform multiple imputations, and then averages the results of multiple imputations to achieve the best result. However, the above methods only perform imputation on scRNA-seq data and cannot conduct dimensionality reduction analysis on the imputed scRNA-seq data simultaneously.

In recent years, with the widespread application of artificial intelligence, machine learning technology has also made great progress. In particular, machine learning techniques have been widely used in biological research. As a common method for data analysis and preprocessing in the field of machine learning, matrix factorization is mainly used for dimensionality reduction, feature extraction and fusion of multidimensional datasets. The most prominent approaches include principal component analysis (PCA) [12], singular value decomposition (SVD) [13], and independent component analysis (ICA) [14], which have been successfully applied in a variety of fields such as transcriptomics [15], proteomics [16], and metabolomics [17,18]. The non-negative matrix factorization (NMF) algorithm [19] is a matrix factorization method with the constraint that all elements in the matrix are non-negative. On the one hand, many large-scale data analysis methods in scientific research need to be effectively processed in the form of a matrix, and the NMF idea provides a new way to process large-scale data. On the other hand, compared with some traditional algorithms, the NMF algorithm has many advantages such as ease of implementation, interpretability of decomposition forms and decomposition results, and less storage space. As NMF is an excellent matrix factorization approach, there are some approaches that use it as a base method to construct imputation schemes for missing values. For example, netNMF-sc [20] uses network regularized non-negative matrix factorization to decompose the expression matrix into two low-dimensional matrices, the cell matrix and the gene matrix. Network regularization allows the two genes connected in the network to have similar expression in the low-dimensional gene matrix, leading to the recovery of missing data in the expression matrix. The CMF [21] method is based on the theory of NMF, and also takes into account cell and gene similarities for the recovery of missing values. Except for a few methods, although NMF-based imputation methods can obtain the low-dimensional representation of samples, they often pay more attention to the clustering analysis results of the imputed high-dimensional data.

**Figure 1:**
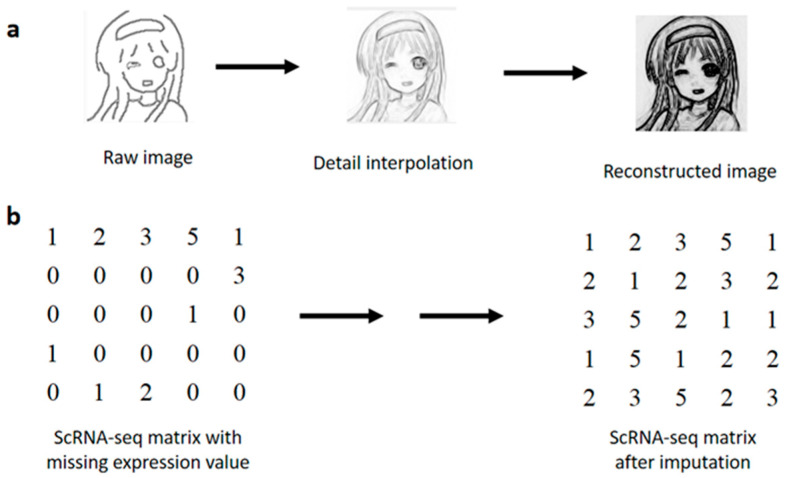
(**a**) An image reconstruction algorithm restores the original information of the image by iteratively adjusting the image matrix. The details in the image are gradually restored, and eventually a complete, detail-rich image is formed. (**b**) A similar strategy can also be used to impute the scRNA-seq expression matrix. ScIRT can reconstruct the scRNA-seq gene expression matrix with missing expression values.

In this study, we present scIRT, a strategy for estimating gene expression loss in scRNA-seq data. Our approach is similar to the image reconstruction method in computer vision, which is mainly based on NMF, starting from the raw single-line image, stepwise detailed imputation and finally obtaining a complete image (Figure 1a). Similarly, we have adapted and applied the original image reconstruction algorithm to gene expression data, completing a stepwise imputation of missing values to obtain complete scRNA-seq data (Figure 1b). Our method is based on the original framework of scHinter [22], which is mainly based on the principle of synthetic minority over-sampling technique (SMOTE) [23], and new constraints are added to each layer of the framework to achieve better convergence results. The advantage of scHinter is that it uses a comprehensive integrated distance measure of multiple distance measures, uses SMOTE for random multi-imputation, and performs random multi-imputation in a multi-layer framework to increase the stability and reliability of the imputation. The distinctive feature of our method is that we have added feature extraction and dimensionality reduction based on scHinter, combining SMOTE and NMF to perform missing data imputation jointly. We have demonstrated the ability of scIRT to recover biological signals on a wide range of scRNA-seq datasets with different sample sizes. The comparative results demonstrate the efficacy of scIRT in the recovery of missing values in datasets. Furthermore, the use of scIRT has been demonstrated to enhance the precision of cell type classification in biological sample data. As a standardized pre-processing instrument, the scIRT provides a flexible and robust approach and can be integrated into a pipeline of other analysis tools for downstream discussion and analysis of scRNA-seq data.

## 2. Results

### 2.1. Overview of scIRT

**Figure 2:**
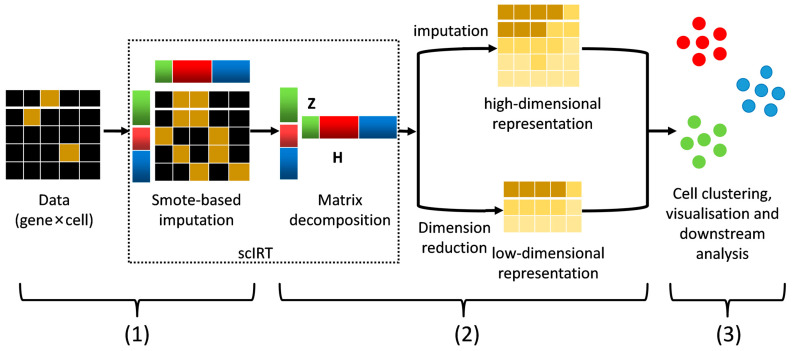
Overview of the workflow for single-cell RNA-seq imputation and cell clustering. These steps include: (1) data imputation based on SMOTE, (2) matrix decomposition based on NMF, (3) using the scIRT tool to output high-dimensional expression matrix and low-dimensional representation matrix for cell clustering analysis as well as other downstream analyses.

The scIRT framework comprises two modules (Figure 2): a data imputation module based on the SMOTE principle, and a matrix decomposition and multi-iterative reconstruction module based on NMF. The scIRT framework stands out due to its high level of integration and convenience, with each module operating independently and interconnected. The input matrix is the gene expression matrix, with the rows representing genes and the columns representing cell samples. The output matrices are the imputed high-dimensional expression matrix and low-dimensional representation matrix of samples for downstream analyses such as cell sample clustering.

The random imputation module employs the SMOTE oversampling method, utilizing the probability density function to assign weights to each layer of cells based on distance. Furthermore, random imputation is performed within a hierarchical framework, whereby cells are progressively assigned greater weights for imputation in a multi-layer manner.

Following imputation, the relevant constraints are added to each layer. These are added by means of matrix decomposition and reconstruction based on NMF, with the aim of making the imputation results of each layer converge. Then, iterative calculations are performed layer by layer. In the current domain, a convergent optimal imputation result is obtained. The estimated expression matrix of scIRT can be used as input to many other scRNA-seq tools for downstream analysis, such as visualization and cell type clustering.

### 2.2. Use scIRT for Dropout Imputation

A ScIRT is a hierarchical framework based on SMOTE random imputation and NMF matrix decomposition. It performs multi-level imputation iterations through stepwise weighting and uses a given cell to impute more similar cells. The Patel-gl dataset [24] is used as an example to illustrate the hierarchical dynamic imputation process. We used the t-SNE visualization technique [25] to show the difference in the number of cells in the gene expression matrix.

**Figure 3:**
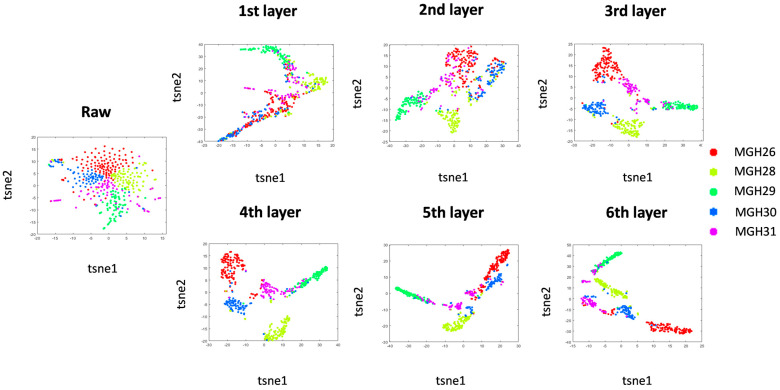
Imputation of data using the hierarchical dynamic scIRT approach. The number of layers is set to 6 according to the average number of cells in the datasets. T-SNE visualization of the original gene-cell expression matrix, and the difference between the cell populations after each round of imputation.

In Figure 3, the initial analysis of the raw data indicated that the cell types were not distinguishable. However, as the number of iterations increased, the different cell types became more clearly defined. Following each random iteration, the differences within the classes became less pronounced, while the separation between the classes increased. After six rounds of imputation, the cells of different types were clearly distinguishable.

**Figure 4:**
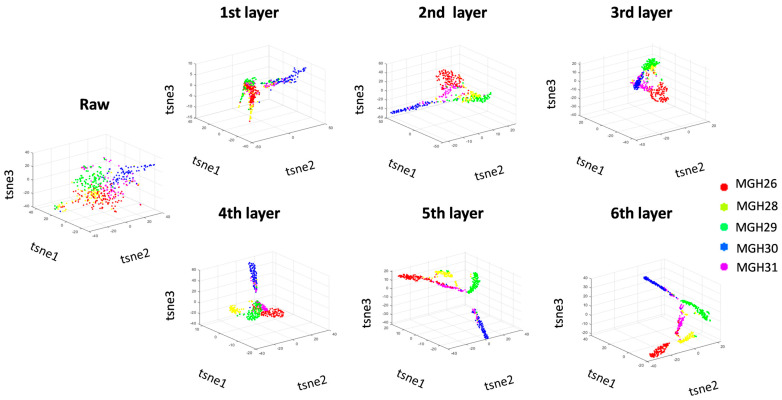
Layered dynamic representation of the imputed data from the scIRT approach using a 3-dimensional model.

In the 3D mode, an increase in the number of iterations leads to an increase in the detection of different cell types (Figure 4). In the spatial domain, it is evident that after each random iteration, the intra-class difference decreases and the inter-class difference increases. Following a six-layer imputation calculation, cells of different types are distinctly separated. The dynamic representation of the data in the spatial domain facilitates a more detailed observation of the data aggregation method of each layer.

As shown in Figure 3 and Figure 4, we use the true labels to classify and label cells, and we can clearly see the dynamic effects of hierarchical clustering of cells in different modes.

### 2.3. ScIRT Optimizes Cell Clustering in High-Dimensional Space

**Figure 5:**
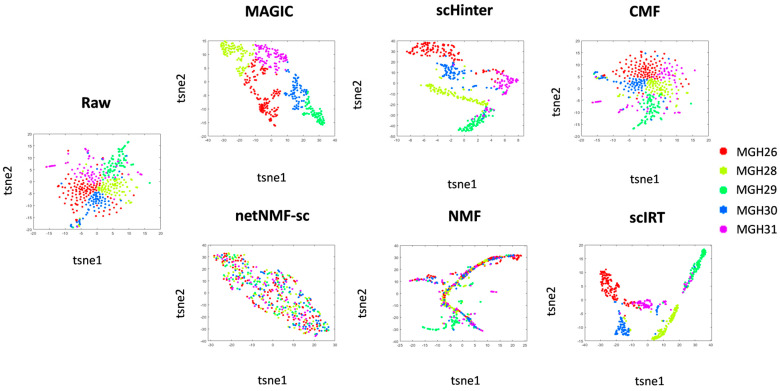
The t-SNE projection of every cell in reduced-dimensional space for the imputed scRNA-seq data by each approach.

Data from Patel-gl was used to validate the imputation performance of each tool, which contains 430 cells of 5 types with a relatively uniform distribution of cell types. We used t-SNE’s dimensionality reduction visualization to analyze the estimated matrix after each tool imputed the data to reflect the differences between cell populations. In a two-dimensional scatter plot, scIRT can better separate different cell types, and the different types are more discrete (Figure 5). It can be seen more intuitively that each tool can gradually group the original scattered points into the form of clusters and clusters, which is more convenient for analysis and use. In comparison with other independent tools, scIRT has been demonstrated to exhibit superior separation effects for diverse cell types in dimensionality reduction visualization images. In summary, the application of scIRT to transform raw data into a more hierarchical format serves to enhance its suitability as a source of input for subsequent analysis.

**Figure 6:**
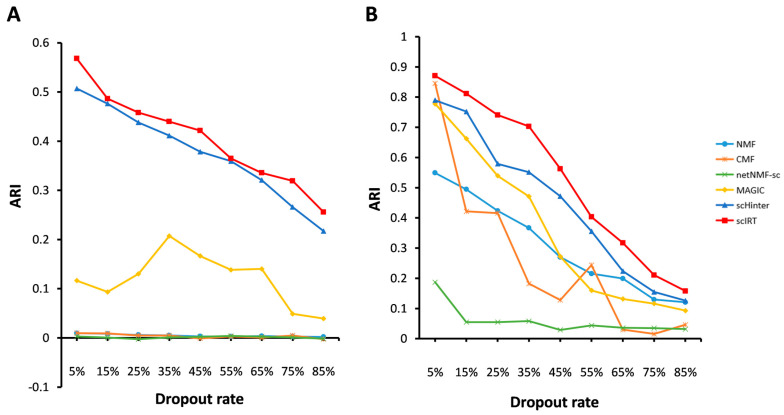
Comparison between scIRT and other imputation tools on simulated scRNA-seq datasets. (**A**) Comparison of ARI metrics between tools on simulated data with different dropout rates produced using the (**A**) Patel-gl dataset and (**B**) Xue-em dataset as a blueprint.

The next stage in evaluating the imputation tools is to compare the clustering metrics of the various tools for data with different missing rates. For this purpose, we still used the Patel-gl dataset. In addition, to improve the validity of the experiments, we added the Xue-em dataset [26] for evaluation. First, we performed an artificial transformation of the Patel-gl dataset. We first counted the total number of elements in the Patel-gl and Xue-em matrices, and then randomly selected a certain percentage of elements and set the corresponding data to zeroes, so that the missing rate in the Patel-gl dataset (Figure 6A) and the Xue-em dataset (Figure 6B) ranged from 5% to 85% (Figure 6). We marked the different tools with six colours and used the Adjusted Rand Index (ARI) metric [27] to measure their performance.

For the Patel-gl dataset, Figure 6A shows that scIRT declines more gently when the deletion rate is between 15% and 45%, and declines a little faster in the interval from 55% to 85%. scHinter shows a gradual downward trend without a flat part. MAGIC also shows a downward trend in general, but an upward trend is observed in some parts of it. The overall performance of NMF, CMF, netNMF-sc is relatively smooth, with no major fluctuations. For the Xue-em dataset, Figure 6B shows that scIRT also performs better in the ARI metrics. Overall, it seems that scIRT outperforms the comparison methods in the ARI metrics for all missing rates. The results conclusively demonstrate that scIRT outperforms other estimation and imputation tools.

### 2.4. Analysis of Co-Expression Structures of Genes

In analyzing co-expression structures within cells, we statistically measured the similarity of the overall structures of two gene expression matrices before and after imputation processing. In this section, we introduced the corr2 two-dimensional correlation coefficient to assess the similarity between two matrices. The corr2 coefficient is a statistical measure of the structural similarity between two two-dimensional matrices. The coefficient ranges from −1 to 1, with values closer to 1 indicating a higher degree of similarity. Essentially, we calculated the Pearson correlation coefficient between the flattened raw and imputed gene expression matrices.

We performed high-variability gene selection on the Xue-em and Leng-h1h [28] datasets to retain the top 2000 ranked genes. We treated the expression data of these selected genes as the raw data. First, we performed clustering calculations on the original Xue-em dataset data, yielding an ARI metric value of 0.3526 and a Silhouette Coefficient (SC) metric [29] value of 0.5588. Clustering using the scIRT-imputed data yielded an ARI metric of 0.6229 and an SC metric of 0.7885. The corr2 metric indicated a similarity of over 0.95 between the matrices before and after imputation. Given this level of similarity, the ARI metric improved by 0.2703, while the SC metric improved by 0.2297. We then performed clustering on the original Leng-h1h dataset, yielding an ARI score of 0.2188 and an SC score of 0.3751. Clustering using the scIRT-imputed data yielded an ARI metric value of 0.4178 and an SC metric value of 0.6344. The corr2 metric indicated a similarity exceeding 0.92 between the pre- and post-imputation matrices. At this level of similarity, the ARI metric improved by 0.1990, while the SC metric increased by 0.2593.

We performed clustering calculations using imputation on the Xue-em dataset and compared the results with those obtained using other imputation tools. The corr2 metrics for NMF, scHinter and scIRT all exceeded 0.95 when measuring the similarity of the two matrices before and after imputation. The ARI metrics obtained by the three imputation tools were 0.3881, 0.3795 and 0.6229, respectively, while the SC metrics were 0.7850, 0.7552 and 0.7885. ScIRT continued to outperform the other two tools. We then performed clustering calculations using imputation on the Leng-h1h dataset. The corr2 metrics for NMF and MAGIC both exceeded 0.95, while those for scHinter and scIRT both exceeded 0.90. The ARI metrics obtained by the four imputation tools were 0.2648, 0.2753, 0.3791 and 0.4178, respectively, with scIRT consistently outperforming the other three tools. Regarding SC metrics, scIRT’s performance was marginally lower than that of scHinter and MAGIC, but still surpassed that of NMF.

The above analysis demonstrates that although the gene expression data after imputation exhibits certain structural differences from the raw cellular information, the use of scIRT to fill this data enhances the clustering efficacy between cells. Our SMOTE-based method do not distort the underlying gene co-expression structure.

### 2.5. ScIRT Improves Cell Clustering Results in Low-Dimensional Space

#### 2.5.1. Impact of Sample Size on Clustering Results

**Figure 7:**
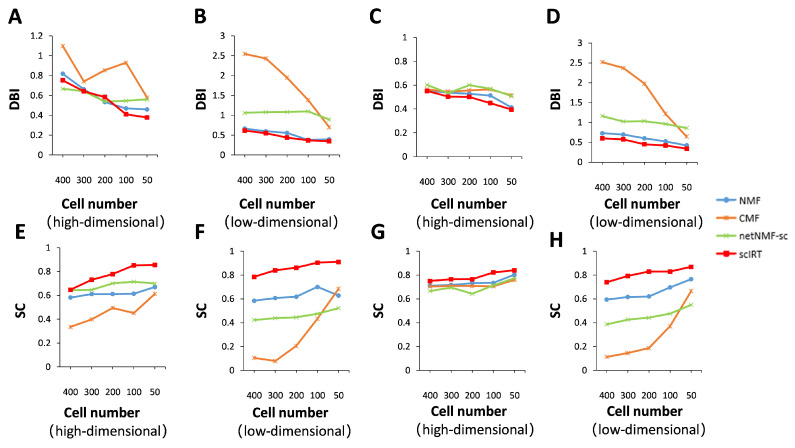
Validating the effect of sample size on clustering results on two publicly available datasets. Performance of the imputation tool on DBI metrics for samples of different sizes in the (**A**,**B**) Li-ct dataset and (**C**,**D**) Darmanis-br dataset based on the high-dimensional and low-dimensional matrices. Performance of the imputation tool on SC metrics for samples of different sizes in the (**E**,**F**) Li-ct dataset and (**G**,**H**) Darmanis-br dataset based on the high-dimensional and low-dimensional matrices.

We investigated the effect of sample size on clustering results. We started with a simple random sampling with put-back on the sample size of the dataset. The number of samples to be taken depends on the sample size of the original dataset, which is 400, 300, 200, 100 and 50. Overall, Figure 7A–D shows the performance of the Davies-Bouldin Index (DBI) metrics [30] calculated based on the high-dimensional and low-dimensional matrices for Li-ct dataset [31] and Darmanis-br dataset [32] using different imputation tools. It can be seen from the graphs that the scIRT’s DBI metrics curve is at its optimum. Figure 7E–H represent the performance of the SC metrics calculated based on the high-dimensional and low-dimensional matrices for Li-ct dataset and Darmanis-br dataset for different imputation tools at different sample sizes, from which we can still see that the metric curve of scIRT is at its best.

These eight charts clearly demonstrate that the DBI and SC metrics are more effective at the 50-sample level than at the 400-sample level. As a whole, the first row of four graphs, with different interpolating tools, shows increasing excellence in the DBI metric as the sample size continues to decrease, and the second row of four graphs, with different interpolating tools, shows increasing excellence in the SC metric as the sample size continues to decrease. For the DBI metric, the curves of different tools generally show a decreasing trend as the sample size decreases, with the curves of NMF and scIRT showing an especially obvious monotonous decrease. For the SC metric, the curves of the different tools show an upward trend as the sample size decreases, while the upward fluctuation of the scIRT curve is more moderate. Figure 7 also demonstrates that the metrics calculated based on the low-dimensional representation matrix of scIRT outperform those calculated based on the high-dimensional matrix on two public datasets. This trend is even more pronounced in the DBI metric. This further suggests that low-dimensional data is more likely to produce favorable clustering outcomes.

#### 2.5.2. Comparison of Cell Clustering Before and After Dimensionality Reduction

Next, we continued to use other two datasets, Patel-gl and Xue-em, to compare cell clustering metrics before and after dimensionality reduction. The Patel-gl dataset contains 5948 genes. The Xue-em dataset contains 14,766 genes. For computational convenience and efficiency, we screened the 14,766 genes in the Xue-em dataset and selected the top 2000 genes with the highest variability. The optimal number of clusters k was selected as 5 and 7, which aligns with the number of categories in the Patel-gl and Xue-em datasets, respectively.

**Figure 8:**
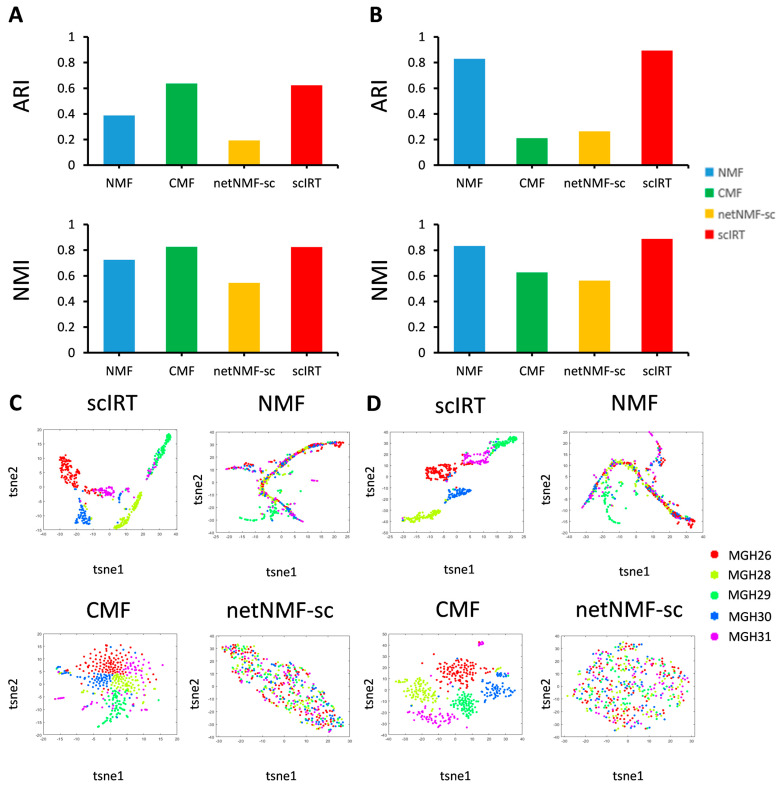
Performance testing of scIRT on two published scRNA-seq datasets. The external clustering metrics, ARI and NMI, were used on the Xue-em dataset to test cell separation and aggregation for (**A**) high-dimensional expression and (**B**) low-dimensional representation. The t-SNE approach projection of cells in reduced-dimensional space on the Patel-gl dataset for (**C**) high-dimensional and (**D**) low-dimensional representation.

ScIRT performed cluster analysis using the low-dimensional representation matrix of the Xue-em dataset and obtained an ARI value close to 0.9, which is 0.27 higher than the ARI value of the high-dimensional expression matrix. Similarly, the NMF method clusters the high-dimensional expression matrix and the low-dimensional representation matrix, yielding ARI values of 0.39 and 0.83, respectively, with a difference of 0.44. The CMF method shows a downward trend in the ARI metric values before and after dimension reduction. However, the netNMF-sc method shows a non-significant increase in the ARI metric values before and after dimension reduction (Figure 8A,B). Compared with high-dimensional expression data, scIRT obtains more highly clustered clusters by using low-dimensional representation data from the Patel-gl dataset, which is also very evident in the graph for the CMF tool (Figure 8C,D).

From the comparison of the two matrices before and after the dimensionality reduction, the clustering metrics computed using the matrices and the performance on the dimensionality reduction scatterplot show that the low-dimensional representation matrix outperforms the clustering effect of the high-dimensional expression matrix to a certain extent. Both matrices can be used to some extent for upstream and downstream analyses in biological studies, with the high-dimensional expression matrix providing complete information on scRNA after imputation and the low-dimensional representation matrix providing better results for clustering analysis.

NetNMF-sc mainly uses a priori information in the form of co-expression or physical interaction networks. However, this may ignore the valid information in the samples and fail to extract a large amount of information, resulting in poor indicator performance. CMF exploits both cell and gene similarities, and such an approach also has some limitations. In contrast, the hierarchical framework embedded in scHinter overcomes the limitations of netNMF-sc and CMF, by allowing multi-layer random imputation, which improves the reliability of obtaining cell similarity. Based on the scHinter framework, scIRT adds new constraints to each layer to achieve better convergence at each layer.

As demonstrated by the analysis of the aforementioned metrics, the scIRT method exhibits superior and more robust performance in a variety of metrics when compared to alternative methods. This analysis is sufficient to demonstrate that the scIRT method possesses a strong ability to recover real gene expression signals. This ability is sufficient to maximize the resolution of different cell types, thereby facilitating and enhancing the efficiency and convenience of downstream bioanalysis.

Next, we added the Leng-h1h dataset [28] to the previous experiment, and a total of three datasets are compared (Table 1 and Table 2). The Leng-h1h dataset contains a total of 19,084 genes. In terms of the number of genes, it is 13,136 genes and 4318 genes more than Patel-gl and Xue-em, respectively, which increases the richness of the test data variety to some extent. We proceeded to analyse the top 2000 highly variable genes selected from the Leng-h1h dataset. In terms of ARI metrics, especially on the Patel-gl and Leng-h1h datasets, scIRT performs the best, all ahead of the other competing methods, but on the Xue-em dataset, scIRT slightly lags behind CMF in terms of ARI and Normalized Mutual Information (NMI) metrics [33] of the complete matrices, but outperforms the other two comparative methods.

In this experiment, we added two clustering internal metrics to verify the effectiveness of scIRT. In this way, we can validate from both internal and external aspects, respectively. The internal metrics do not require the use of cluster labels and assess the quality of the clusters based solely on the characteristics of the data itself. Such metrics usually focus on the compactness within clusters and the separation between clusters. After clustering the high-dimensional expression matrix and low-dimensional representation matrix of the Xue-em dataset, scIRT achieved SC values of 0.7885 and 0.8228, respectively. These performances are superior to those of the comparison methods. ScIRT performs comparably to other methods when using the Leng-h1h dataset. However, when using the Patel-gl dataset, NMF performs better and exhibits the best SC and DBI metrics in the clustering calculation of the low-dimensional representation matrix.

From the above analysis of the metrics, it can be seen that scIRT maintains a higher and more robust performance in the four metrics of ARI, NMI, DBI, and SC compared with other methods. The above analysis is sufficient to prove that scIRT has a strong ability to recover the real gene expression signals. This ability maximizes the resolution of different cell types and makes downstream bioanalysis more convenient and efficient.

From the point of view of the global data of the scIRT approach, out of a total of 12 data pairs, 10 sub-matrices (83%) outperform the high-dimensional expression matrix for the metrics, while 2 data pairs (17%) show a decrease in the performance of the metrics. Overall, scIRT leads the overall comparison metrics by 71%, CMF by about 12.5%, as does NMF, and netNMF-sc by about 4%.

### 2.6. Performance Evaluation of Runtime and Memory Consumption

We compared the computation times of NMF, CMF, netNMF-sc, scHinter, MAGIC, and scIRT. Here, we selected the top 100, 200, 500, 1000, and 2000 highly variable genes for the Patel-gl dataset (Table 3). At the number of genes greater than or equal to 1000, scIRT is faster than netNMF-sc, and comparable to the time of CMF, scHinter.

We further observed the performance of scIRT in terms of memory consumption (Table 4). As in the same experiment, we chose five sets of highly variable genes with numbers of 100, 200, 500, 1000, 2000 to test and calculated the variance of the memory consumption data in five sets of the data, which are 364, 9774, 271, 8060, 2935, and 35, respectively. ScIRT has the smallest variance in comparison with other methods.

We calculated the memory consumption percentage using the memory consumption calculated in the previous experiment, with the total amount of memory (8065 MB). This result demonstrates more clearly that scIRT does not show a significant change in memory consumption when the number of genes increases (Table 5).

## 3. Discussion

Due to the extremely high loss rate and sparsity of scRNA-seq data, it is necessary to develop an approach to correct the noise and reduce the loss rate of data prior to downstream analysis of scRNA-seq data. We proposed an integrated framework, scIRT, to impute missing values in scRNA-seq data. ScIRT is a highly compact and flexible framework consisting of two modules, including a data imputation module based on SMOTE and a matrix decomposition module based on NMF. The imputation approach of scIRT is well-suited for random imputation of hierarchical models with limited datasets. We have conducted a comprehensive research evaluation of the performance of scIRT on various scRNA-seq datasets and compared it with MAGIC, scHinter, CMF, netNMF-sc, NMF and raw data. These comparative approaches cover both NMF-based and non-NMF-based methods, which gives a certain degree of comprehensiveness. The results show that scIRT has superior and more robust performance compared to other competing approaches on several metrics.

Currently, due to the limitations of single-cell sequencing technology, dropout events are still the main problem of scRNA-seq data. ScRNA-seq data, which contain a large amount of cellular variability, have severely limited the development of downstream biological analysis. It is very critical to recover these data. MAGIC uses the shared information of similar cells to impute data based on the idea of thermal diffusion. Unlike MAGIC, netNMF-sc mainly uses a priori information from co-expression networks, which may ignore valid information in the samples and fail to extract a large amount of valid information. CMF exploits two different kinds of similarities of cells and genes at the same time, and this method also suffers from the limitation of interfering with each other. Therefore, the imputation performance of these methods did not improve significantly with increasing sample size. scHinter uses an ensemble distance metric for imputation, but has certain limitations in metric weighting. To overcome this limitation, scIRT uses image reconstruction technology to impose certain constraints on the computational results of each layer, which has a convergence effect on the imputation results.

ScIRT employs a hierarchical framework to progressively include more cells to perform imputation, creating a multi-layer random imputation to better separate different types of cells. Moreover, the integrated framework of scIRT enables the extraction of information from data with increasing sample sizes, thereby enhancing the robustness of the imputation results.

It should be emphasized that scIRT is an efficient imputation tool that can analyze missing data more effectively. There are statistical tools for modelling specific dropout cases, such as MAGIC, scHinter, netNMF-sc, CMF, and NMF. However, the imputation performance of these tools is not very efficient for missing data. We have developed scIRT to estimate dropout events and have shown that using the scIRT imputation module can make imputation of missing data more efficient.

In order to enhance the compatibility of scIRT with a range of data in future, it is imperative to address the following issues. Primarily, the current range of data that scIRT is able to utilize is limited, thus necessitating the development of additional modules and more complex models to facilitate its adaptation to a wider variety of data types. To this end, concerted efforts are underway to enhance the adaptability of scIRT to diverse data types. The present version of scIRT is implemented in MATLAB (R2018b), and efforts are being made to extend this data analysis tool to a range of platforms.

In terms of scalability, when handling large-scale datasets, the next phase of our project will enhance scIRT to ensure it continues to operate efficiently when processing large-scale datasets. We will integrate novel frameworks that emerge from the latest advancements in computational biology and single-cell research [34,35,36,37] with scIRT to improve its performance. In the future, our research will focus on advanced encoding strategies, the predictive modelling of nucleotide modifications and the analysis of RNA-based regulatory elements [38,39,40,41], with the aim of enriching the background research on scIRT.

## 4. Materials and Methods

### 4.1. Public scRNA-Seq Datasets

To study the scIRT approach, we used datasets with different numbers of cells, genes and cell populations. These limited sample datasets can be used to evaluate the performance of different imputation tools (Table 6). The five datasets are as follows: (1) Patel-gl dataset (GEO; GSE57872) [24], (2) Xue-em dataset (GEO; GSE44183) [26], (3) Li-ct dataset (GEO; GSE81861) [31], (4) Darmanis-br dataset (GEO; GSE67835) [32], (5) Length-h1h dataset (GEO; GSE64016) [28].

### 4.2. Aggregate Distance Metrics to Calculate Intercellular Similarity

To calculate similarity between cells, we commonly employ multiple distance metrics. However, selecting the optimal metric proves highly challenging, as different metrics learn similarity through distinct data features, exhibiting significant variation across datasets [42]. ScHinter proposes an ensemble distance metric that integrates multiple distance measures to obtain a ranking of cellular similarity. ScIRT employs a similar ensemble distance approach to scHinter for calculating cellular similarity.

For the pair of cells i and j, their dissimilarity is calculated based on the nth distance metric ${\mathrm{DIS}}_{\mathrm{ij}}^{n}$ as follows (Equation (1)):

(1) $${\mathrm{DIS}}_{\mathrm{ij}}^{n}\;=\;\mathrm{distance}\;({\mathrm{CELL}}_{i},{\;\mathrm{CELL}}_{j})$$

For each cell in the gene expression matrix, the distance matrix is obtained for the nth distance metric (${\mathrm{DIS}}^{n}$), where each row represents the distance between that cell and the others in the matrix. The distance value vector for each row (e.g., cell i) is then converted into an ordinal variable (Equation (2)).

(2) $$R_{i*}^{n}=\mathrm{rank}\;({\mathrm{DIS}}_{i*}^{n})$$

In the aforementioned equation, ${\mathrm{DIS}}_{i*}^{n}$ denotes the dissimilarity between cell i and the other cells in the gene expression matrix, calculated using the nth distance metric. Meanwhile, $R_{i*}^{n}$ represents the corresponding ordinal value. For a gene expression matrix comprising y cells, the ith element of the ordinal vector is set to 1 since cell i is most similar to itself. In the event that cell j is identified as the next cell most similar to cell i, then the jth element of the ordinal vector is designated as 2, and the process is repeated accordingly. This yields the ordinal matrix $R^{n}$, where each row represents the distance between the ordinal variable of a given cell and those of the other cells in the gene expression matrix. Subsequently, the n ordinal matrices are combined into a set matrix using an integrated distance method (Equation (3)).

(3) $$M=\sum_{n}\left(t_{n}\times R^{n}\right)$$

where $t_{n}$ denotes the weight assigned to the nth distance metric. The scIRT approach assigns a weight value of 1. Researchers may assign different weight values according to their specific requirements.

### 4.3. Random Cell Imputation Based on SMOTE

The random imputation method employed in this study is derived from SMOTE [23]. When considering a gene expression matrix comprising y cells and s genes, cell i is represented by a vector $a_{i}$ consisting of s elements, signifying that this cell possesses s genes. The similarity between cell i and other cells in the gene expression matrix is computed via integrated distance. To characterize the similarity between cell i and another cell, a random weight coefficient f is assigned to each cell. The imputed value of $a_{i}$ may be updated according to Equation (4).

(4) $$a_{i}^{*}=a_{i}+\sum_{j=1}^{y}f_{j}(a_{j}{-a}_{i})$$

The weight f represents a trade-off between the intrinsic information of the original cell and the external information from other similar cells, determining the extent to which external information is utilized during the imputation process. Equation (4) can be expressed in an alternative form as follows:

(5) $$a_{i}^{*\;}=\left(1\;-\sum_{j=1}^{y}f_{\mathrm{ij}}\right)a_{i\;}+\;[f_{i1},f_{i2},f_{i3},\cdots ,f_{\mathrm{iy}}]\left[\begin{array}{c} \begin{array}{c} a_{1}\\ a_{2}\\ a_{3} \end{array}\\ \cdots \\ a_{y} \end{array}\right]$$

In the aforementioned expression, $\sum_{j=1}^{y}f_{\mathrm{ij}}$ equals the parameter RE. When RE = 0, it signifies that no imputation processing is performed, and all information for cell i is retained. When RE = 1, it signifies that only the minimal information encoded by cell i in $\left(f_{ii}\times a_{i}\right)$ is retained, whilst the majority of cell i’s updated information derives from other cells within the gene expression matrix. In our scIRT research, we set RE to 0.9. Other researchers may adjust this parameter accordingly to suit their specific requirements.

In general, missing information in target cells within the expression matrix may be compensated for by obtaining data from similar or neighboring cells. Typically, the closer a target cell is to another cell, the greater its weight is assigned. However, it is not possible to accurately measure the similarity between two cells through expression alone. In datasets with limited sample sizes, learning from similar or neighboring cells can result in overfitting. In scIRT research, we use a method similar to scHinter to enhance the accuracy of measuring cell similarity by randomly assigning weights to similar cells. This approach simultaneously mitigates overfitting. Under this strategy, the weight $f_{j}$ for a similar cell $a_{j}$ even if it is the closest to the target cell $a_{i}$ is not necessarily the maximum value. Instead, it is randomly selected from the weights assigned to similarly ranked cells.

In this approach, we employed the probability density function of the geometric distribution [22] to compute the numerical value of the weight f (Equation (6)).

(6) $$P\left(A=x\right)={(1-p)}^{x-1}p$$

In Equation (6), the range of values for x comprises positive integers such as 1, 2, 3, and so forth.

The gene expression matrix comprises y cells, with NUM predicted clusters. The parameters p in the geometric distribution may be determined through the average cell count (expected value) and shrinkage coefficient shrink for each cluster [22].

(7) $$p=\frac{\mathrm{NUM}}{\mathrm{shrink}\;\times \;y};\;\;E\left(A\right)=\frac{1}{p}$$

### 4.4. Multi-Layer Random Imputation Approach

Within the multi-layer stochastic imputation framework of scIRT, the first two layers adhere to the scHinter paradigm. Subsequently, a new layer is introduced to perform matrix decomposition on the imputed data. The aim of this process is to produce high-dimensional and low-dimensional expression matrices for clustering purposes.

The first layer of this multi-layer stochastic imputation framework employs a geometric distribution probability density function (p = 1/2) to assign weights to two cells belonging to the same category as the target cell (Equation (8)). For cells not belonging to the same category as the target cell, weights are sequentially assigned from the lower region of the probability density curve based on their similarity ranking relative to the target cell. This random weight allocation is essentially represented by Equation (4). The second layer uses a probability density function of p = 1/4, allocating most of the weights to the four cells that belong to the same category as the target cell. This process is repeated until the layer contains all cells from the target cell’s category (Equation (8)). The top-level stratum retrieves weights from a flat probability density curve (p = NUM/y (Equation (7)). This means that all cells similar to the target cell receive an equal weighting.

(8) $$A^{*}=[W\left(f_{\mathrm{NUM}}\right)\times \ldots \times W\left(f_{4}\right)\times W\left(f_{2}\right)]\times A$$

In Equation (8), the $W(f_{n})$ represents the weight matrix for the (log2n)th layer. NUM denotes the estimated average number of cells within each cluster, determining the upper limit of layers in the multi-layer framework (log2(NUM)). Here, we employ a base-2 logarithm to determine the number of layers. ScIRT introduces a new layer beyond the original scHinter framework structure, decomposing the interpolated matrix A* to yield $A_{h}$ and $A_{l}$.

(9) $$\left[A_{h},\;A_{l}]=\mathrm{NMF}(A^{*}\right)$$

In Equation (9), $A_{h}$ denotes the high-dimensional representation matrix, while $A_{l}$ denotes the low-dimensional representation matrix. Researchers may select between them according to their specific requirements.

### 4.5. Data Model and Iterative Update Rules of NMF

The paper by Lee and Seung provides a reasonable solution for NMF [19]. They decompose the original matrix A into two factors: $Z\;\in {\;R}^{u\times k}$ and $H\;\in \;R^{k\times v}$, and try to approximate a factorization (Equation (10)):

(10) $$A^{+}\;\approx \;Z^{+}H^{+}$$

The approximate representation of the three matrices is shown in Equation (11).

(11) $$\left[\begin{array}{ccc} a_{11}^{+} & \ldots & a_{1v}^{+}\\ \vdots & \ddots & \vdots \\ a_{u1}^{+} & \ldots & a_{\mathrm{uv}}^{+} \end{array}\right]=\left[\begin{array}{ccc} z_{11}^{+} & \ldots & z_{1k}^{+}\\ \vdots & \ddots & \vdots \\ z_{u1}^{+} & \ldots & z_{uk}^{+} \end{array}\right]\times \left[\begin{array}{ccc} h_{11}^{+} & \ldots & h_{1v}^{+}\\ \vdots & \ddots & \vdots \\ h_{k1}^{+} & \ldots & h_{kv}^{+} \end{array}\right]$$

The objective function of the NMF algorithm has two expressions. The first objective function corresponds to the Euclidean distance [43] (Equation (12)):

(12) $$O_{1}\;=\;{\left\|\;A\;-\;\mathrm{ZH}\;\right\|}^{2}=\sum_{u,v}{\left({\;A}_{\mathrm{uv}}-\;{\left(\mathrm{ZH}\right)}_{\mathrm{uv}}\right)}^{2}$$

The second objective function corresponds to the K-L divergence [44] (Equation (13)):

(13) $$O_{2\;}=\;D\left(\;A\left\|\mathrm{ZH}\right.\;\right)\;=\sum_{u,v}\left({\;A}_{\mathrm{uv}}-\log\frac{A_{\mathrm{uv}}}{{\left(\mathrm{ZH}\right)}_{\mathrm{uv}}}-A_{\mathrm{uv}}\;+\;{\mathrm{ZH}}_{\mathrm{uv}}\;\right)$$

The iterative update rules can be divided into two types according to the different noise distributions. Assume that the noise of the matrix follows a Gaussian distribution [45]. The iterative update rules can be expressed as follows (Equations (14) and (15)):

(14) $$Z_{\mathrm{uk}}\leftarrow Z_{\mathrm{uk}}\frac{{(AH^{T})}_{\mathrm{uk}}}{{(\mathrm{ZH}H^{T})}_{\mathrm{uk}}}$$

(15) $$H_{\mathrm{kv}}\leftarrow H_{\mathrm{kv}}\frac{{(Z^{T}A)}_{\mathrm{kv}}}{{(Z^{T}\mathrm{ZH})}_{\mathrm{kv}}}$$

Assume that the noise of matrix the follows the Poisson distribution [46]. The update iteration rules can be expressed as follows (Equations (16) and (17)):

(16) $$Z_{\mathrm{uk}}\leftarrow Z_{\mathrm{uk}}\frac{\sum_{v}\left[H_{\mathrm{ku}}A_{\mathrm{uv}}/{\left(\mathrm{ZH}\right)}_{\mathrm{uv}}\right]}{\sum_{v}H_{\mathrm{kv}}}$$

(17) $$H_{\mathrm{kv}}\leftarrow H_{\mathrm{kv}}\frac{\sum_{u}\left[{\;Z}_{\mathrm{uk}}A_{\mathrm{uv}}/{\left(\mathrm{ZH}\right)}_{\mathrm{uv}}\;\right]}{\sum_{u}Z_{\mathrm{uk}}}$$

### 4.6. Combined Application of NMF and SMOTE

ScIRT leverages the fundamental framework of scHinter, incorporating NMF matrix factorization in the step c and adding step d below where NMF is utilized once more, yielding the results: high-dimensional expression matrix and low-dimensional representation matrix. As shown in Algorithm 1:

- The similarity between cells is computed using a voting-based set distance metric, and several commonly utilized distances are integrated into a collective solution.
- Random imputation employs the SMOTE oversampling strategy, assigning random weights to cells based on the integrated comprehensive distance.
- The hierarchical framework is implemented in a multi-layer approach, progressively incorporating additional cells for random imputation. NMF matrix factorization is employed to analyze the results of random imputation at each layer. Through multiple iterations, the root mean square residuals derived from the actual and predicted values of random imputation results at each layer are minimized. The imputation outcomes are computed sequentially for each layer.
- The outcomes are refined by applying NMF matrix factorization once more using the imputation results from the final layer. Through this process, both the high-dimensional expression matrix and its corresponding low-dimensional representation matrix are derived through calculation.

**Algorithm 1.** Application framework of the scIRT algorithm**Input:** gene dataset (data), weight (wt), number of categories (nc). **Step 1.** [total_rank] = distance_consensus (data,wt); **Step 2.** [m,n] = size (data); **Step 3.** mean_number = floor (log2(m/nc)); **Step 4.** times = 2^(1: mean_number)^; **Step 5.** expectation of each cluster (eec) = floor (m/nc × 0.9); **Step 6.** for i ← 1 to length (times).do data_new = SMOTE (data,total_rank,m,eec)[matrix1, matrix2] = NMF (data_new) data_new_NMF = matrix1 × matrix2end **Step 7.** [u,v] = NMF (data_new_NMF); **Step 8.** high-dimensional matrix = u × v, low-dimensional matrix = u. **Output:** high-dimensional expression matrix, low-dimensional representation matrix. Note: The first step is the distance_consensus function, which implements step a. In the second step, the size function mainly calculates the size of the matrix. Here, m represents the number of rows and n represents the number of columns. The floor function and log2 function in the third step are used to find the integer in the negative infinity direction and calculate the logarithm with base 2, respectively. The floor function in step five has the same effect as step four. The length function in step six calculates the length of the vector. Step six and implements steps b and c. Step seven implements step d.

### 4.7. Comparative Methodology and Metrics

In this experiment, four categories of clustering metrics are used for evaluation, including DBI [30], SC [29], ARI [27], and NMI [33], which mainly cover the internal and external categories of clustering. The methods selected for our comparison mainly comprise NMF-based methods, as well as other approaches.

Methods based on NMF matrix decomposition: Non-negative matrix factorization (NMF) is an unsupervised learning algorithm that decomposes a matrix into a matrix of non-negative weight coefficients and a basis matrix. It is commonly used in feature learning, image analysis and topic recognition. CMF is a method based on matrix decomposition that takes into account both cell and gene similarities when analyzing data. NetNMF-sc is a method for utilizing gene co-expression information in the imputation and dimensionality reduction of scRNA-seq data.

Other methods: MAGIC is a method for sharing information across similar cells through data diffusion, which is used to remove noise from cellular expression matrices and populate missing transcripts. ScHinter is a method that employs voting-based ensemble distances and performs stochastic imputation using the SMOTE oversampling technique. ScHinter also incorporates a hierarchical frame structure to enhance the accuracy of small-sample imputation.

## Figures and Tables

**Table 1:** Imputation performance results for NMF, CMF, netNMF-sc and scIRT based on Xue-em, Patel-gl, and Leng-h1h datasets (ARI, NMI).

Data	Method	Xue-em		Patel-gl		Leng-h1h	
		ARI	NMI	ARI	NMI	ARI	NMI
High- dimensional matrix	NMF	0.3881	0.7259	0.0190	0.1087	0.2648	0.2792
	CMF	**0.6364**	**0.8259**	0.0105	0.0696	0.3275	0.3634
	netNMF-sc	0.1920	0.5455	0.1555	0.1941	0.0235	0.0288
	scIRT	0.6229	0.8248	**0.4820**	**0.6725**	**0.4178**	**0.4488**
Low- dimensional matrix	NMF	0.8309	0.8338	0.0080	0.0662	0.3650	0.3708
	CMF	0.2109	0.6259	0.5516	0.6937	0.3824	0.3995
	netNMF-sc	0.2630	0.5622	0.1149	0.1882	0.0201	0.0306
	scIRT	**0.8942**	**0.8881**	**0.7636**	**0.7527**	**0.5791**	**0.5415**

Note: The highest score for the ARI and NMI metric is the best performance, and the best performances are shown in bold.

**Table 2:** Imputation performance results for NMF, CMF, netNMF-sc and scIRT based on Xue-em, Patel-gl, and Leng-h1h datasets (DBI, SC).

Data	Method	Xue-em		Patel-gl		Leng-h1h	
		DBI	SC	DBI	SC	DBI	SC
High- dimensional matrix	NMF	0.5010	0.7850	0.5736	**0.9601**	1.3470	0.2935
	CMF	1.1125	0.4250	1.1067	0.7266	1.7318	0.2097
	netNMF-sc	0.9551	0.4543	1.4327	0.3359	0.5768	**0.6799**
	scIRT	**0.4902**	**0.7885**	**0.4919**	0.7690	**0.5300**	0.6344
Low- dimensional matrix	NMF	0.5079	0.7834	**0.3264**	**0.9582**	0.8560	0.5743
	CMF	**0.4047**	0.7816	3.6242	0.0517	3.5051	0.0283
	netNMF-sc	1.0646	0.3910	1.4147	0.3054	1.6794	0.3093
	scIRT	0.4492	**0.8228**	0.7340	0.6983	**0.5044**	**0.7716**

Note: The minimum score for the DBI metric is the best performance, the maximum score for the SC metrics is the best performance, and the best performances are shown in bold.

**Table 3:** Time consumption (seconds) of different approaches on Patel-gl dataset.

Tool	Genes = 100	Genes = 200	Genes = 500	Genes = 1000	Genes = 2000
NMF	0.37	0.39	0.40	0.53	0.82
CMF	6.74	8.05	12.61	24.49	65.67
netNMF-sc	14.66	20.03	46.41	165.13	464.49
scHinter	22.10	23.42	22.41	22.48	23.02
MAGIC	3.93	4.15	3.80	3.83	4.56
scIRT	23.78	22.42	24.47	25.86	26.17

Note: Time run tests were conducted on the same computer [Intel(R) Core (TM) i5-4590 CPU@3.30 GHz, 4 CPU cores].

**Table 4:** Memory consumption of different approaches on Patel-gl dataset.

Tool	Genes = 100	Genes = 200	Genes = 500	Genes = 1000	Genes = 2000
NMF	4785	4800	4807	4830	4828
CMF	4647	4669	4685	4667	4886
netNMF-sc	4793	4794	4806	4826	4783
scHinter	4544	4710	4742	4778	4706
MAGIC	4777	4836	4874	4769	4887
scIRT	4579	4587	4582	4591	4593

Note: The RAM capacity of the computer is 8065 MB, obtained from MATLAB (R2018b)’s ‘feature(’memstats‘)’ command with the ‘Physical Memory (RAM)’value obtained by the parameter ‘total’ in MATLAB (R2018b)’s ‘feature(‘memstats’)’ command. Or the value obtained from the parameter ‘info.total’ in Python (3.7.4)’s ‘info = psutil.virtual_memory()’ command.

**Table 5:** Percentage of RAM consumption of different approaches on Patel-gl dataset.

Tool	Genes = 100	Genes = 200	Genes = 500	Genes = 1000	Genes = 2000
NMF	59.33	59.52	59.60	59.89	59.76
CMF	57.61	57.89	58.09	57.87	60.58
netNMF-sc	59.43	59.44	59.59	59.84	59.31
scHinter	56.34	58.40	58.80	59.24	58.35
MAGIC	59.23	59.96	60.43	59.13	60.59
scIRT	56.78	56.88	56.81	56.92	56.95

Note: The memory consumption percentage is obtained by dividing the memory consumption by the total amount of memory.

**Table 6:** Overview of datasets used in scIRT.

Data No.	Dataset	No. of Genes	No. of Cells	Species	Sample	Reference
1	Patel-gl	5948	430	Human	Glioblastomas	[24]
2	Xue-em	14,766	29	Human	Embryos	[26]
3	Li-ct	57,241	561	Human	Colorectal tumors	[31]
4	Darmanis-br	22,088	466	Human	Brain	[32]
5	Leng-h1h	19,084	247	Human	H1 hESC	[28]

## Data Availability

The original contributions presented in this study are included in the article. Further inquiries can be directed to the corresponding author.
